# Application of an analytical approach to characterize the target strength of ancillary pelagic fish species

**DOI:** 10.1038/s41598-023-42326-4

**Published:** 2023-09-13

**Authors:** Antonio Palermino, Andrea De Felice, Giovanni Canduci, Ilaria Biagiotti, Ilaria Costantini, Michele Centurelli, Iole Leonori

**Affiliations:** 1 CNR-National Research Council, IRBIM-Institute for Marine Biological Resources and Biotechnologies, Largo Fiera Della Pesca, 1, 60125 Ancona, Italy; 2 Alma Mater Studiorum, Università di Bologna, Via Zamboni, 33, 40126 Bologna, Italy

**Keywords:** Marine biology, Mathematics and computing

## Abstract

The lack of data on the species-specific Target Strength (TS) on ancillary species limits the application of acoustic surveys in assessing their abundance and distribution worldwide. The TS values of *Scomber colias* and *Trachurus mediterraneus* in use in the Mediterranean Sea rely on studies conducted on other species in the Atlantic and Pacific oceans. Nevertheless, the application of backscattering models offers the possibility to overcome the absence of empirical data handling the parameters that most affect the TS. X-ray scans were performed on 82 specimens to get digital representations of the swimbladder and the fish body which were used as input for the application of the Kirchhoff Ray Mode model to measure the TS as a function of frequency and tilt angle. The morphometric differences between the two species produced divergent relative frequency responses and broadband TS patterns. Moreover, comparing the results with one ex-situ experiment, we found a good agreement considering a mean tilt angle of − 10°, standard deviation = 12°. Our results provide the first theoretical insights into the use of backscattering models as a tool to distinguish between species in the Mediterranean Sea by acoustic method, increasing the knowledge of the acoustic reflectivity of ancillary species.

## Introduction

Atlantic chub mackerel* (Scomber colias)* and Mediterranean horse mackerel (*Trachurus mediterraneus)* are two pelagic species found in the temperate waters of the Mediterranean and Atlantic Seas. They play an important ecological role in the pelagic niche since they are mainly plankton and small fish feeders that contribute to linking the lower and the higher level of the trophic chain^1–3^. However, they are generally considered ancillary species in the Adriatic Sea^4^. In the Mediterranean Sea, they have low economical value and they mostly represent bycatch and discard of the small pelagic fishery^5,6^. Yet, in recent years *S. colias* and *T. mediterraneus* assumed certain importance as commercial food sources, as demonstrated by the expanding landings. *S. colias* catches, which showed an increasing trend from 2005 to 2019 in the Adriatic Sea and the same trend was registered from 2010 to 2019 in the entire Mediterranean Sea^7^. Otherwise, in some areas of the Eastern Atlantic, it became a target species, marked by an exponential increase in landings during the last 15 years^8^. *T. mediterraneus* accounts for around 1.3% of all landings in the Mediterranean, with total catches of about 45,000 tons in 2016–2018^9^, and a substantial portion of landings in the southwestern Mediterranean^6^ and the Adriatic Sea^10^.

The aforementioned data underlines the importance that the species covered by this study is progressively gaining in Mediterranean waters. Nevertheless, only recently, a stock assessment was performed on Mediterranean horse mackerel^4^ thanks to the data collected during the regular acoustic survey carried out annually in the Adriatic Sea within the framework of the EU Mediterranean International Acoustic Survey (MEDIAS) project^11^, whereas, in the Atlantic Ocean the stocks of these species are assessed yearly based on acoustic surveys accomplished by the International Council for the Exploration of the Sea (ICES). The authors note that survey and commercial catch data on these species are often not reliable for different reasons, such as the lack of geographical coverage, misidentification of species, and gear selectivity^8,12^. When acoustic survey time series are available, the poor and heterogeneous knowledge of the species-specific target strength (TS) of these secondary species does not allow us to make reliable estimates of species biomass^8^ which is particularly valuable in the case of the application of harvest control rules in the management plan. Both STECF and ICES stress the importance of monitoring the status of the *Scomber* and *Trachurus* genus in the Mediterranean Sea and Atlantic Ocean.

Acoustic surveys have the potential to provide reliable fishery-independent data since they are viewed as one of the highly effective approaches for assessing the distribution and abundance of pelagic species^13^. However, the conversion of volume backscattering strength, provided by the surveys, to an absolute biomass estimate requires knowledge of the species-specific acoustic backscattering cross-section. This is expressed in terms of target strength relation: TS = 10log_10_(σ/4π) in which sigma is the amount of incident wave reflected by the cross-section of a single target and includes the scattering properties of the species convolved with behaviour^13–15^. In-situ and ex-situ methods are suitable for the measurement of the species-specific TS in the natural environment or controlled conditions and are currently considered the best method to compute the TS to convert volume backscattering strength into biomass from acoustic survey^16–19^. However, only the use of backscattering models allows us to predict theoretical backscatter from accurate measurement and setting of organism anatomy, material properties, swimbladder morphology, tilt angles, and frequencies^20^. TS is affected by acoustic frequency, fish body length, orientation (tilt angle), depth and physiological factors^21,22^. Furthermore, the backscatter is mostly attributable to the dimensions and shape of the swimbladder, which is responsible for up to 95% of the backscatter of a fish^15^. Therefore, in fish species identification and assessment using scientific echosounders, the knowledge of the cross-sectional area, volume and tilt angle of the swimbladder is of primary importance.

The first published models conventionally modelled the swimbladder as a simple geometrical shape such as a sphere, a finite cylinder or a prolate spheroid/ellipsoids solving the backscatter through analytical solutions^23,24^. Successively, more sophisticated models called approximate analytical models based on the approximation of the swimbladder shape were pursued by sectioning it into a compound of finite cylinders solved by Kirchhoff Approximation model (KA), Deformed Cylinder Model (DCM) and Kirchhoff Ray approximation Mode model(KRM)^25,26^. Subsequently, complex finite surface elements were solved using numerical models such as the Boundary Element Method (BEM), the Fourier matching model (FMM) and theFinite Element Method (FEM)^27,28^. All these models are still in use, each having advantages and constraints, and the choice of model depends on the target shape, the tilt angle range investigated, data availability and power computer availability^20,29^. Numerical models are computationally demanding and require detailed measurements of swimbladder and fish body characteristics. They account for the finer morphometric variation on the surface resulting in more precise and accurate measurements of acoustic reflectivity, which can help to draw broadband frequency patterns^30^. Conversely, analytical models approximate complex swimbladder shapes by solving the backscatter from one or more simplified geometric figures but, they do not require super-computer computations and high-resolution measurements^31^. Generally, there is a good agreement between models and empirical experiments^31–33^, but some authors found significant differences between models, in-situ and ex-situ experiments^33–35^.

With a continuously growing interest in the use of broadband in acoustic surveys, backscattering models give insight into the feasibility of implementing broadband techniques for species identification purposes. The recent commercial availability of echosounders capable of both narrowband and broadband is leading to a shift in acoustics data collection in favour of broadband^36,37^. One of the main advantages is the increase in near-frequency resolution that improves the characterization and classification of acoustic targets^38^. The use of broadband pulses gives fisheries acoustics scientists the possibility to distinguish fish of different sizes belonging to the same species^30^. However, to the best of the author's knowledge, a single work was published on broadband acoustics response of fish in the Mediterranean Sea^39^, where the multi-frequency backscatter still represents a good tool for species discrimination^40^. Moreover, backscattering models have never been used on the species dealt with in this study.

In this paper, we collected digital images of fish anatomy through X-ray scans to develop approximate analytical models in order to investigate the swimbladder morphology and compute the species-specific TS functions of *S. colias* and *T. mediterraneus* in the Adriatic Sea. Results were compared with ex-situ experiments conducted on the same species in the same area. We used the Kirchhoff ray mode model (KRM) to study the variation of TS as a function of tilt angle, frequency and fish length. In particular, we focused attention on the development of species-specific TS vs Total length (TL) function. Moreover, by considering different tilt angle intervals, we proposed a species-specific relative frequency response (RFI) and broadband backscatter curve. Results were compared between species providing preliminary evidence on the possible application of RFI and broadband pulses for species discrimination purposes in the Adriatic Sea.

## Materials and methods

### Fish sample

**Figure 1:**
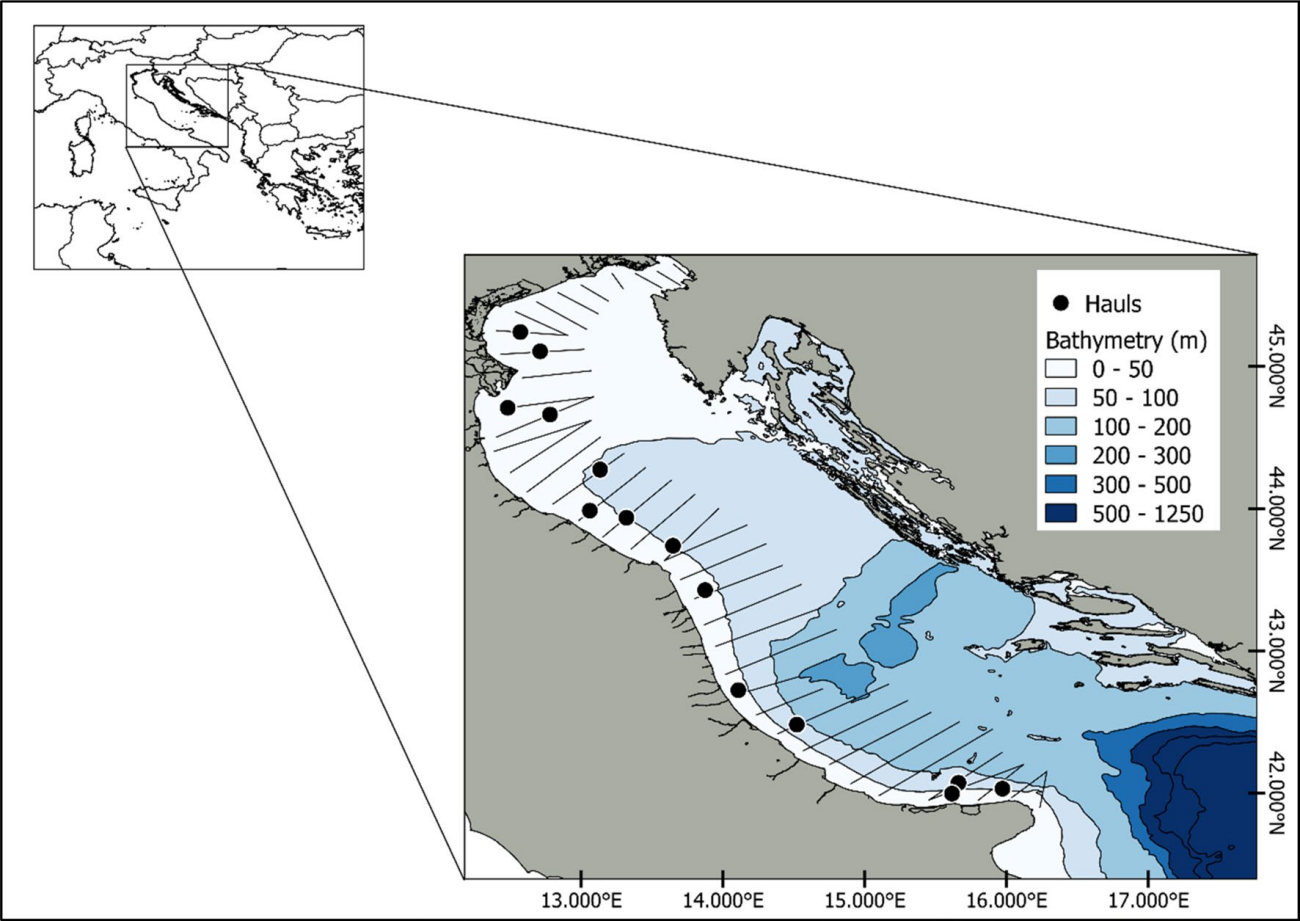
Net sampling positions (black dots). The figure also reports the transect plan followed during the 2020 and 2021 MEDIAS survey in the North Adriatic Sea (GSA 17).

Fish were collected in the Adriatic Sea during the 2020 and 2021 MEDIAS survey carried out between June and July by the Acoustics Group of CNR-IRBIM of Ancona on board of R/V G. Dallaporta. The fishing operations were undertaken with a mid-water trawl characterized by an 18 mm cod-end mesh size equipped with SIMRAD’s FX80 trawl sonar to monitor the behaviour of the net. The net was cast at a seabed depth ranging from 25 to 90 m at around 4 knots for ~ 30 min. Before each trawl, water temperature and salinity were collected using a CTD probe (SEABIRD 911 PLUS) to compute the sound speed in the water. The sites of the hauls where the fish were collected are shown in Fig. 1. A data collection plan was developed as follows: once on board, active and healthy fishes were immediately transferred and held in a 200-L tank with running seawater on board for at least 4 h, 12 h when possible. After this period of acclimation surface pressure, the fish that exhibited normal swimming behaviour were anaesthetized in the tank mixing a path of 9:1 ethanol with 4 ppm of clove oil to avoid the possible release of gas during the successively freezing operation^41^. All fishes were frozen as soon as possible at − 20° in plastic bags. In order to reduce the influence of depth pressure, hauls carried out at the surface or at low depth have been primarily selected for specimen collection. The study complies with the Italian animal research legislation (D. Lgs. N. 26 of 04/03/2014).

### Swimbladder measurements

**Figure 2:**
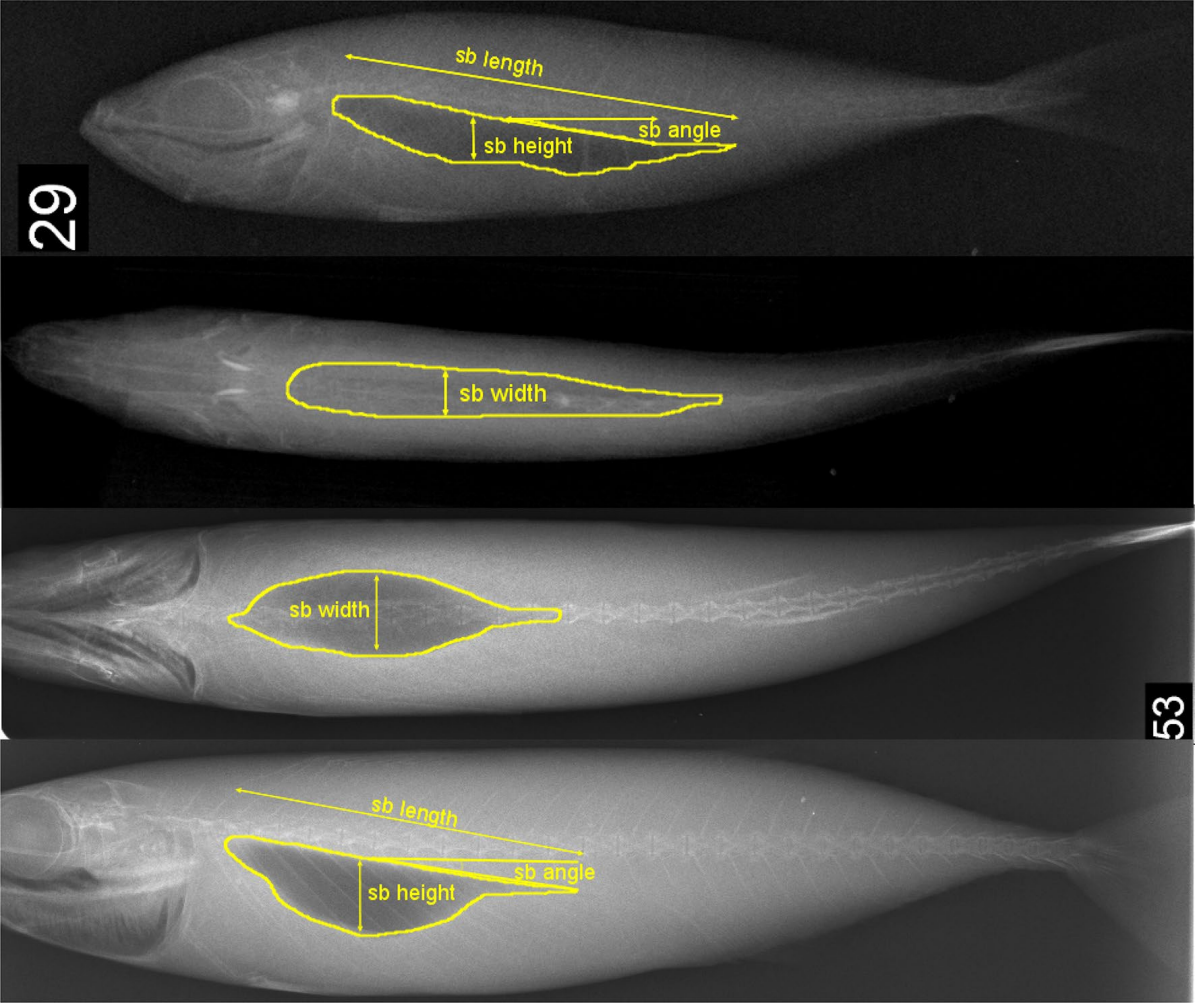
Soft X-ray lateral and dorsal radiographs of *T. mediterraneus* (**a**) and *S. colias* (**b**). Swimbladder borders are shown with yellow lines, while the arrows depict the swimbladder measurements.

Specimens were defrosted and scanned with an RT 400 HF/TS X-ray at veterinary facilities. The fishes were placed at about 1 m from the X-ray source on a detector plate with known dimensions. In each session from 1 to 5 specimens were radiographed on dorsal and lateral view to collect two-dimensional projection images of the swimbladder (Fig. 2). Different trials with other fishes of the same dimensions collected during the survey were carried out before image acquisition, in order to adjust instrument settings to guarantee maximum resolution. The exposure and current were 2 mA/s while the voltage was set at 52–65 (kVp)^32,42^. A metal ichtyometer was placed in each scan for image calibration purposes.

DICOM image files from X-ray scans were processed using ImageJ software. The images that presented an inflated and intact swimbladder were adjusted to improve the contrast between the swimbladder and the fish's body and when necessary the fish was rotated to get a horizontal sagittal axis. Thereafter, a grayscale threshold was set to automatically trace the boundary of the fish body and the swimbladder. Whereas the greyscale threshold was unable to accurately detect the swimbladder boundaries (especially on dorsal view), they were traced manually. We collected dorsal surface—swimbladder length (sbl), defined by the distance between anterior and posterior margins—swimbladder height (sbh), defined by the maximum thickness in the lateral aspect—swimbladder width (sbw), defined by maximum thickness in the dorsal aspect and swimbladder tilt angle (sbϴ), defined as the tilt between the centreline and the straight line crossing the latter, as shown in Fig. 2. Next, from the traced dorsal surface, we computed the mean cross-sectional area, while the volume was obtained following the formula: $V= 4\pi /3 (sbl/2)(sbh/2)(sbw/2)$. All measurements are reported in Fig. 2. Measurement results were plotted against the TL through a linear regression model. Afterwards, a t-test was performed to compare the swimbladder morphologies of the two species. Finally, a log-regression model was set on both species to investigate the growth curve of the mean cross-sectional area against TL. Next, each fish was placed in a Cartesian plane and ${x}_{j}$, ${ z}_{j}$ and ${w}_{j}$ coordinates, where ${x}_{j}$ and ${z}_{j}$ are the upper and lower coordinates along the longitudinal axis and ${w}_{j}$ is the width of the body on dorsal view, were collected, initially each 10 mm for the fish body and 5 mm for the swimbladder and subsequently each 1 mm for both.

### Backscatter modelling and data analysis

Coordinates obtained from X-ray image processing of fish with inflated and intact swimbladder were used as input parameters for the application of the Kirchhoff ray mode model. Kirchhoff-Ray approximation is used for computing the scattering of finite-length cylinders, summing coherently the scattering from consecutive and potentially offset gas-filled (swimbladder) and fluid-filled (fish body) stacked cylinders, which gives a 3D representation of the fish and the swimbladder. A correction for the tapers at each end of the elements is included to improve the accuracy of the model^43^. Typical acoustic fish parameters required by the model and water parameters, computed on the basis of the environmental data collected during the survey with a CTD (SEABIRD 911 PLUS) probe, are given in Table 1. KRMr package^44^ was used to apply the KRM model on fish and swimbladder shapes. An analytical model on a theoretical sphere was used as a benchmark for KRM backscatter results. A theoretical sphere along with the fish body and swimbladder shape collected for a *S. colias* specimen were employed to assess the KRM performance by comparing the results obtained from coarse and refined slice thickness in a range between ʎ/10 and 2 mm.

**Table 1:** Acoustic parameters used for KRM computations.

Model parameters	Values	Source
Speed of sound in water (${\mathrm{ms}}^{-1}$)	1509	CTD measurements
Speed of sound in fish body (${\mathrm{ms}}^{-1}$)	1570	Gauthier and Horne^29^
Speed of sound in swimbladder (${\mathrm{ms}}^{-1}$)	345	Clay and Horne^25^
Density of the water (${\mathrm{kgm}}^{-3}$)	1026	Gauthier and Horne^29^
Density of fish body (${\mathrm{kgm}}^{-3}$)	1070	Gauthier and Horne^29^
Density of the swimbladder (${\mathrm{kgm}}^{-3}$)	1.24	Clay and Horne^25^

Moreover, the ensemble influence of slice thickness and tilt angle was performed. Finally, the TS of a three-dimensional prolate spheroid with semi-major ($sbl/2$) and semi-minor ($\sqrt {(sbh} *sbw/4)$) axis modelled following the size of a typical swimbladder size of a small *S. colias* specimen, was computed by solving the Kirchhoff—Helmholtz integral equation in the Acoustic modules of COMSOL Multiphysics 6.0 software through the Finite Element Method (FEM) in broadband, in order to validate the result of the KRM model. More details on the FEM model and formula can be found in supplementary materials.

The backscattering cross section was computed as a function of the tilt angle strictly within a range between 65° and 115° because, as demonstrated by Macaulay et al.^45^, at a high off-broadside tilt angle the KRM model becomes not accurate. The corresponding backscattering cross section in the linear domain (σ_bs_) was averaged for each chosen Gaussian tilt angle interval and then logarithmically transformed to TS values. The following tilt angles distribution was chosen to represent near-normal and abnormal swimming behaviour of fish: 90° ± 5°; 90° ± 10°; 90° ± 20°. The other tilt angle intervals are intended to represent the increasing swimming-orientated direction of fish. A tilt angle of 90° with a standard deviation of 10 and 20 was set as suggested by Membiela and Dell’Erba^46^ for fish spread at different depths. Then we added another tilt angle interval to compute the TS considering an abnormal fish swimming behavior. The mean tilt angle of 101° with a standard deviation of 12° was set adding the mean and standard deviation of the swimbladder related to the fish body angle computed during the swimbladder measurements. Corresponding mean TS values for each tilt angle interval were then regressed in function of fish TL using the standard model: $TS = m log L + b$ and the model proposed by Foote (1987): $TS= 20 log L + {b}_{20}$. Mean ${\sigma }_{bs}$ from the normal tilt angle interval (88° s.d. 13°) and at a broadside angle at 38 kHz were related to the results of the other discrete frequencies usually used during acoustics surveys (70, 120, 200 kHz) to compute the relative frequency response following the formula: ${r}_{i}$(f) = ${\sigma }_{i}$(f)/${\sigma }_{i}$(38)^47^.

## Results

### Swimbladder morphology

A total of 82 specimens divided as follow were collected: 25 Atlantic chub mackerels (TL size range, 11–33.7 cm), and 57 Mediterranean horse mackerels (TL size range, 11.2–27.7 cm). The size range obtained was roughly representative of the one commonly found in the Adriatic Sea. 5 specimens for each species out of the 82 fish subjected to X-ray scan showed a deflated swimbladder. The data analysis on the remaining animals gave the values reported in Table 2. The wide fish size range, especially for *S. colias*, occasioned a high gap between minimum and maximum values of swimbladder measurements. Despite the similar mean TL, the swimbladder of *T. mediterraneus* is on average 20 mm longer, 1 mm smaller and 2.3 mm less wide compared to *S. colias*, which in turn leads to different volume and area.

**Table 2:** Morphological characteristics of fish body and swimbladder of *T. mediterraneus* and *S. colias*.

Measure	*T. mediterraneus*	*S. colias*				
	Mean	Min	Max	Mean	Min	Max
Total length (cm)	15.4	11.2	23.1	14.4	11.1	33.7
Sb length (mm)	50	35.4	77.7	30	6.7	93.8
Sb width (mm)	4.8	2.7	7.4	7.1	3.6	22.4
Sb height (mm)	4	2.3	6.9	5	2	18.7
Volume (mm^3^)	562	150.7	1833	2027.7	74.2	20,628
Area (mm^2^)	198.1	68.7	475.4	184	45.3	1132.5
Sb ϴ (°)	10.9	5.54	14.8	11.4	6.34	14.3

**Figure 3:**
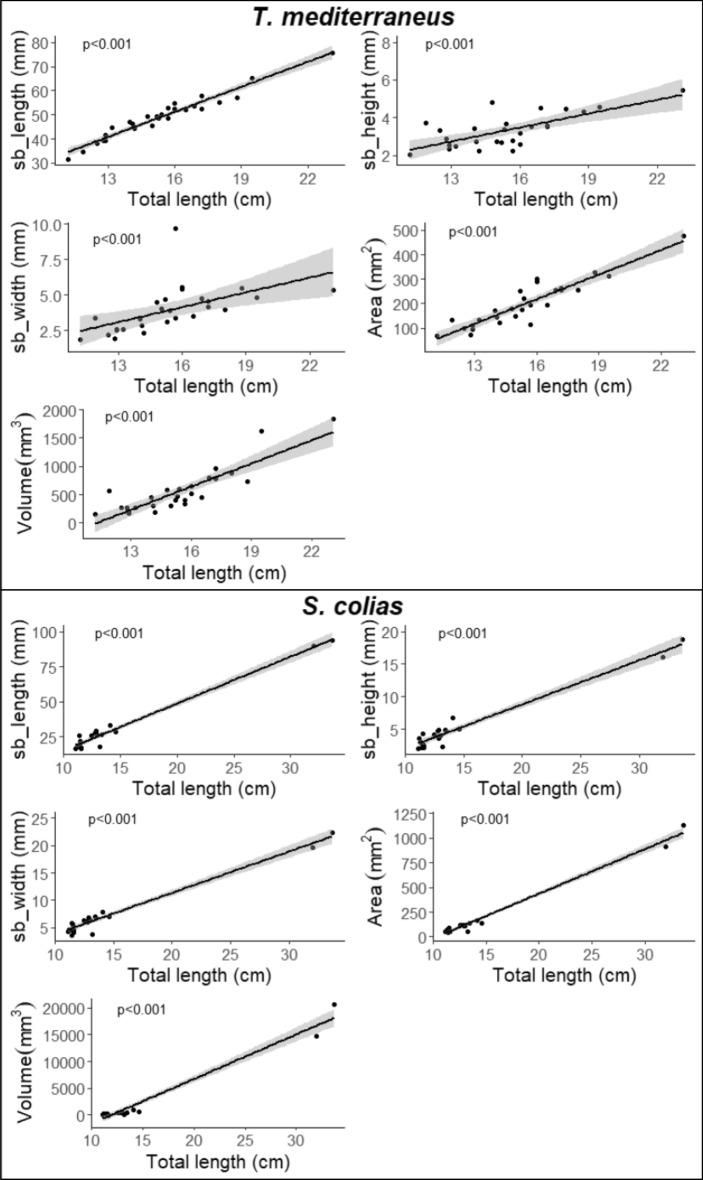
Linear relationships between swimbladder length, height, width, area and volume and total length of *T. mediterraneus* (top) and *S. colias* (below). The shadow areas express the 95% confidence intervals.

**Figure 4:**
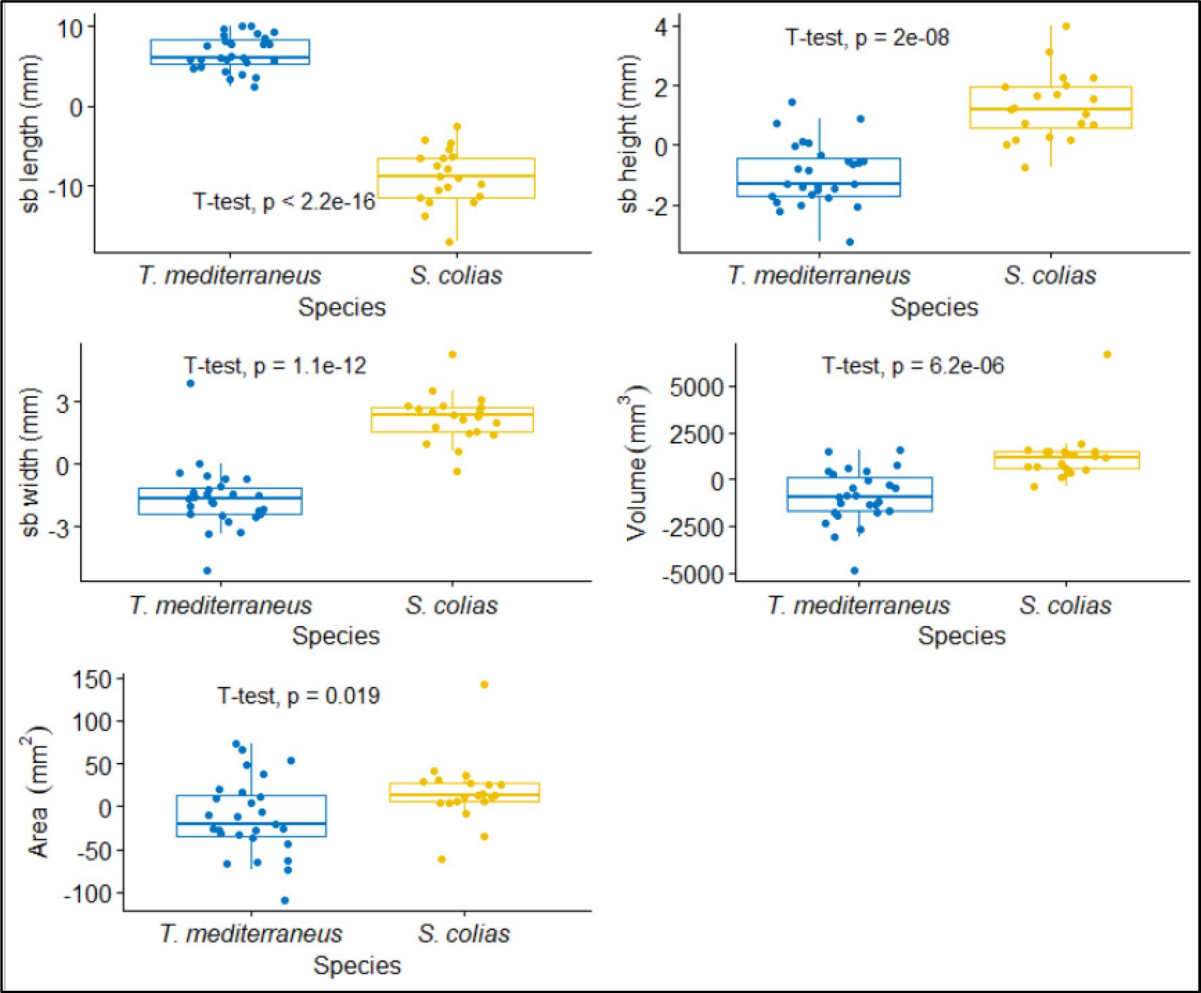
Comparisons between dimensionless swimbladder morphological characteristics of *T. mediterraneus* and *S. colias*. The p-value resulted from the Student t-test are depicted. The x axes report the values of the residuals resulting from the linear regression models shown in Fig. 3. The distribution of data is illustrated by a boxplot showing the medians (horizontal lines), percentiles (box borders), and 5–95% percentiles (vertical lines).

Swimbladder length, height, width, volume and area increase proportionally to the total length. The regressions depicted in Fig. 3 clearly show a significant positive relationship characterized by a high level of significance (*p* ≤ 0.001) and a good fit of all models (r^2^ ≥ 0.95). Therefore, in order to remove the effect of length, the residuals of the models were kept as relative swimbladder sizes for comparison between the two species' morphologies. Once the length effect was removed, the t-test revealed high divergence in swimbladder length, height, width, and volume (*p* ≤ 0.001 in each case) while a lower but still significant difference characterized the area (*p* ≤ 0.05). Overall, the swimbladder of *T. mediterraneus* appears elongated, pear-shaped and slightly compressed laterally with a thin posterior end that increases towards the head up to a wide frontal region. The haemal spines, clearly visible inside the cavity, generally make ripples on the swimbladder surface. Conversely, *S. colias* displays a spherical-like swimbladder characterized by narrow anterior and posterior regions defined by large width and height proportionally to the length as shown in Fig. 4.

### KRM accuracy

**Figure 5:**
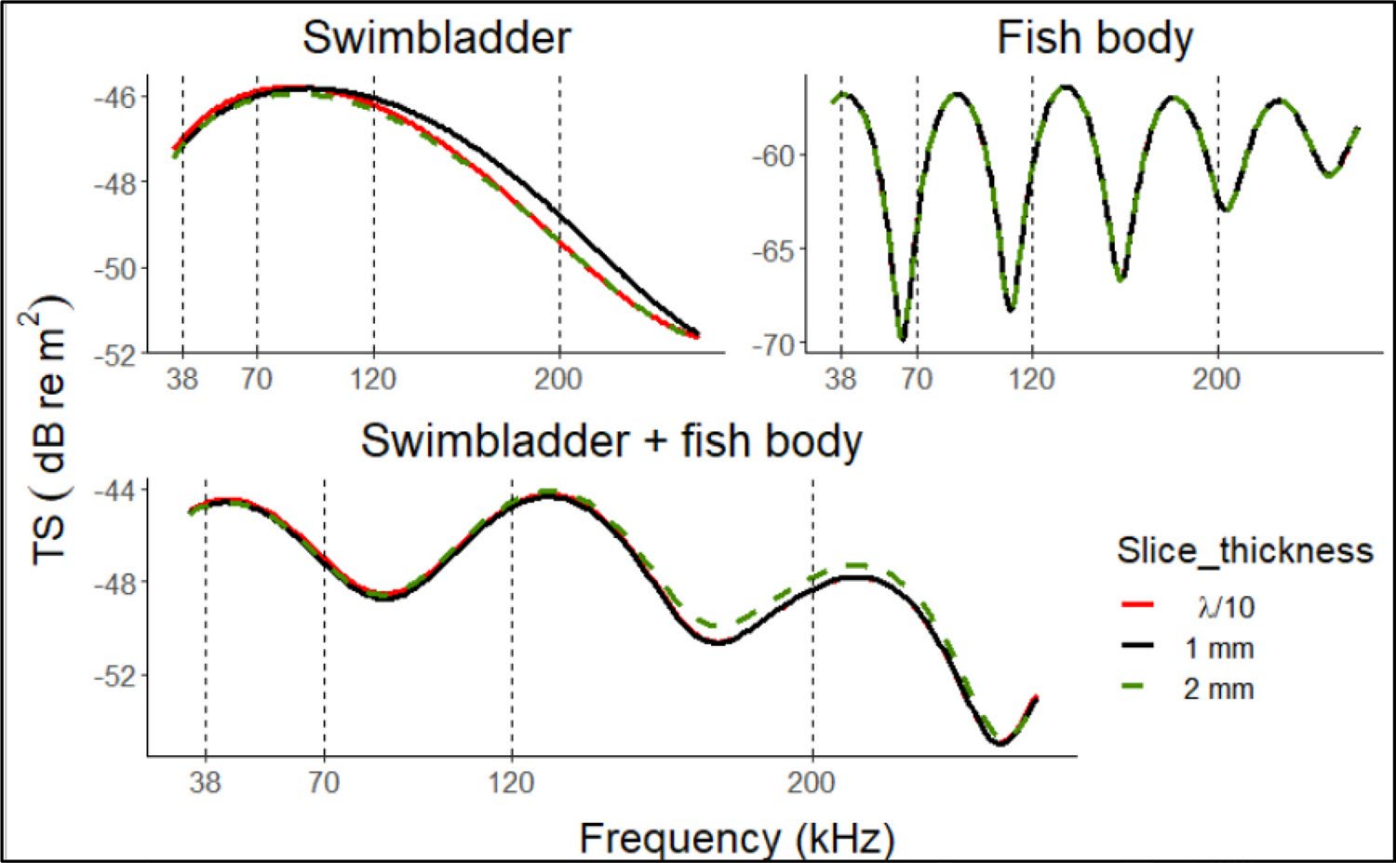
TS versus frequency calculated using the KRM model on swimbladder only (top left), fish body only (top, right) and the whole fish (below) of a *S. colias* specimens of 12 mm TL. The three colors point out the size of cylinder (ʎ is computed on 200 kHz). The vertical dashed lines indicate the reference frequencies more frequently used in fisheries acoustics.

**Figure 6:**
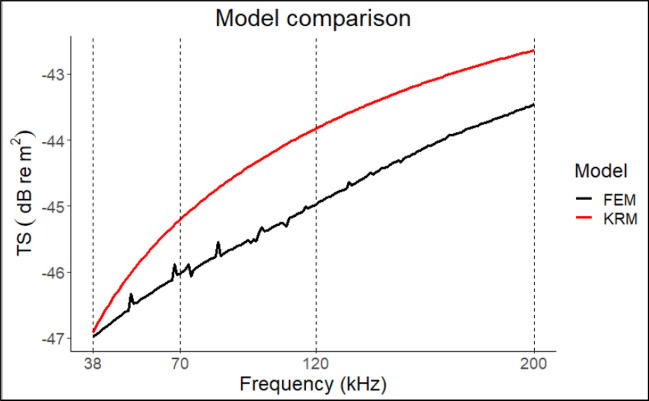
The TS vs frequency of a prolate spheroid of 0.01 m semi-major axes and 0.002 m semi-minor axes calculated through the KRM model (in red) and the FEM model (in black). The vertical dashed lines indicate the reference frequencies more frequently used in fisheries acoustics.

The KRM model employed in this study shows a good agreement with the analytical model (see supplementary Figure S1). Figure 5 shows the results of the KRM model in broadband considering a broadside angle of a single 11.5 cm specimen of *S. colias* of as an example. A small fish was preferred, as a trade-off between representation and computational time needed to solve the equations. The model was applied to refined and coarse measurements, firstly only on the swimbladder (length = 19 mm) and later by removing the swimbladder from the fish body and finally summing the TS backscatter from the union. The results showed differences up to 0.4 dB in the swimbladder. Notably, over 120 kHz the TS curve obtained with a slice thickness of 2 mm is closer to the one obtained with ʎ/10. No differences were detected for the fish bodywhile considering the whole fish, TS resulted higher for the 2 mm slice thickness at high frequencies. TS curve has undertaken detectable changes among cylinder size only at steeper negative angles (− 25°) (more details in supplementary Figures S2–S3). Conversely, significant differences up to 10 dB on the backscatter were detected between 2 and 1 mm slice thickness computations for a sphere of 0.2 mm radius, while there were any variations between ʎ/10 and 1 mm except for the fluid sphere over 220 kHz (see Supplementary Figure S4 for further details). For these reasons, in the subsequent analysis, we used a slice thickness of 1 mm as a good trade-off both for swimbladder and fish body measurements. The KRM model was also tested on a prolate-spheroid against the FEM model (Fig. 6). The prolate spheroid semi-major and semi-minor axis were defined based on the swimbladder dimensions of a small specimen (semi-major axes = 0.015 m; semi-minor axes = 0.002 m) from one species covered by this study, in order to limit the CPU necessary for computations.

The simulations indicated that the model agreed within 1 dB along the frequencies spectrum from 70 to 200 kHz and 0.5 dB between 38 and 70 kHz.

### Target Strength analysis

Table 3 shows TS-TL function results based on five tilt angle distributions. *b* values of the standard model were lower than the *b*_20_ values except for a mean tilt angle of 101° with a standard deviation of 12°, which does not fit well into the linear regression for *T. mediterraneus* (*r*^2^ ~ 0.1).

**Table 3:** TS-TL standard model and with slope forced to 20 by tilt angle distributions.

Tilt angle (°)		TS = *m* log* l* + *b*				TS = 20 log *l* + *b*_20_		
Mean	St. dev	*m*	*b*	*s.e*	*r*^2^	*b*_20_	*s.e*	*r*^2^
*T. mediterraneus*								
90	5	21.3	− 65.88	1.2	0.62	− 64.27	1.19	0.62
90	10	24.6	− 70.12	1	0.75	− 64.51	1	0.73
90	20	25	− 71.58	0.78	0.84	− 65.48	1.63	0.80
101	12	8.88	− 56.52	1.43	0.14	− 70.04	1.6	0.1
88	13	26	− 71.65	0.86	0.78	− 64.4	0.95	0.76
*S. colias*								
90	5	24.65	− 71.25	1.25	0.88	− 66	1.34	0.82
90	10	26	− 73.2	1.26	0.88	− 66.44	1.47	0.82
90	20	25.72	− 74.06	1.3	0.89	− 67.58	1.48	0.84
101	12	14.2	− 62.54	1.16	0.74	− 69.43	1.38	0.85
88	13	26.9	− 74.48	1.3	0.89	− 66.65	1.59	0.83

Standard model slope (m), conversion parameters *b* and *b*_20_, standard error (s.e.) and R^2^ are shown. The values are expressed in dB re 1 m^2^.

**Figure 7:**
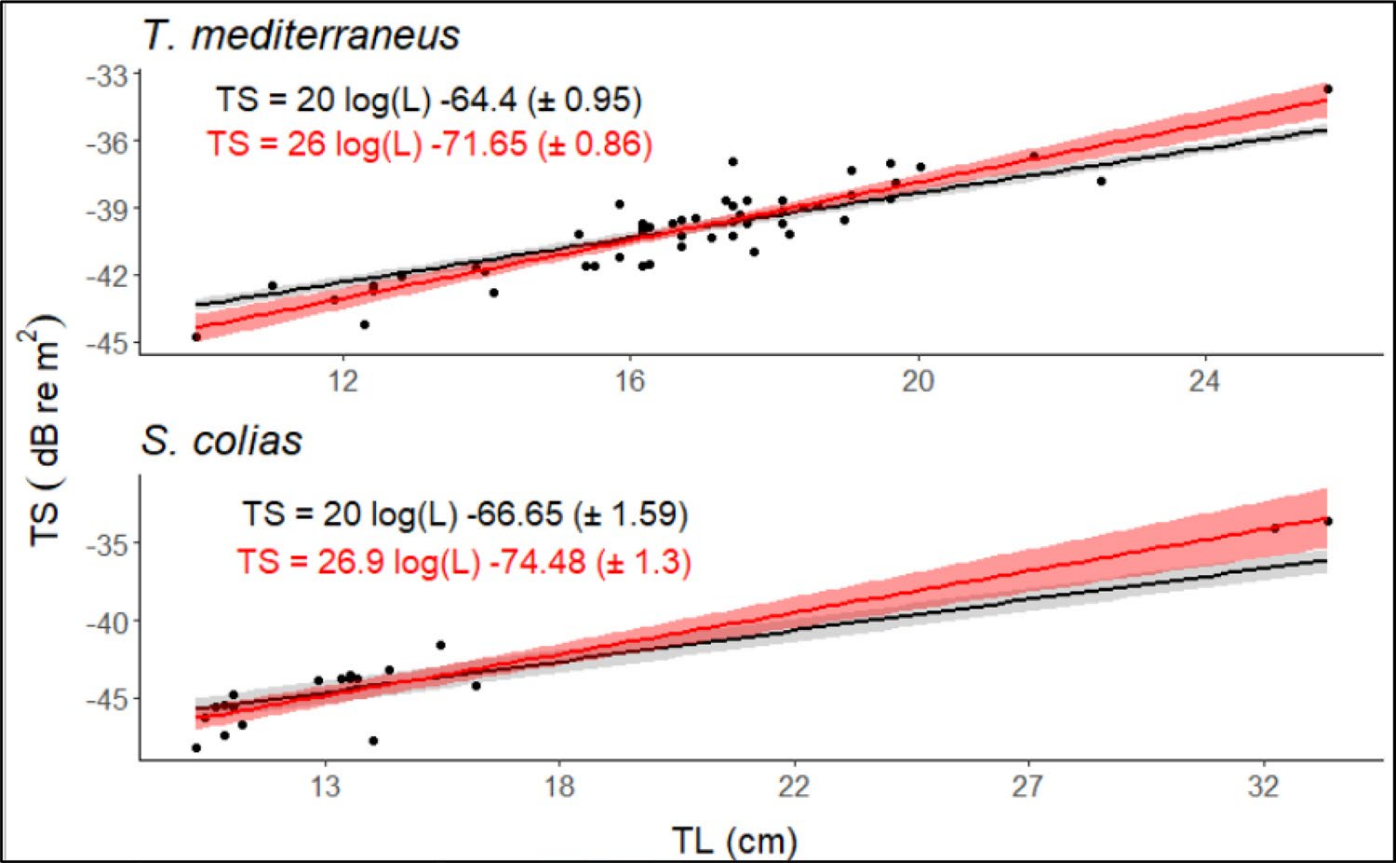
Target strength vs total length relationship with a mean fish tilt angle of 88° s.d. 13°. The standard model regression is shown in red, and in black the model with the slope forced to 20.

**Figure 8:**
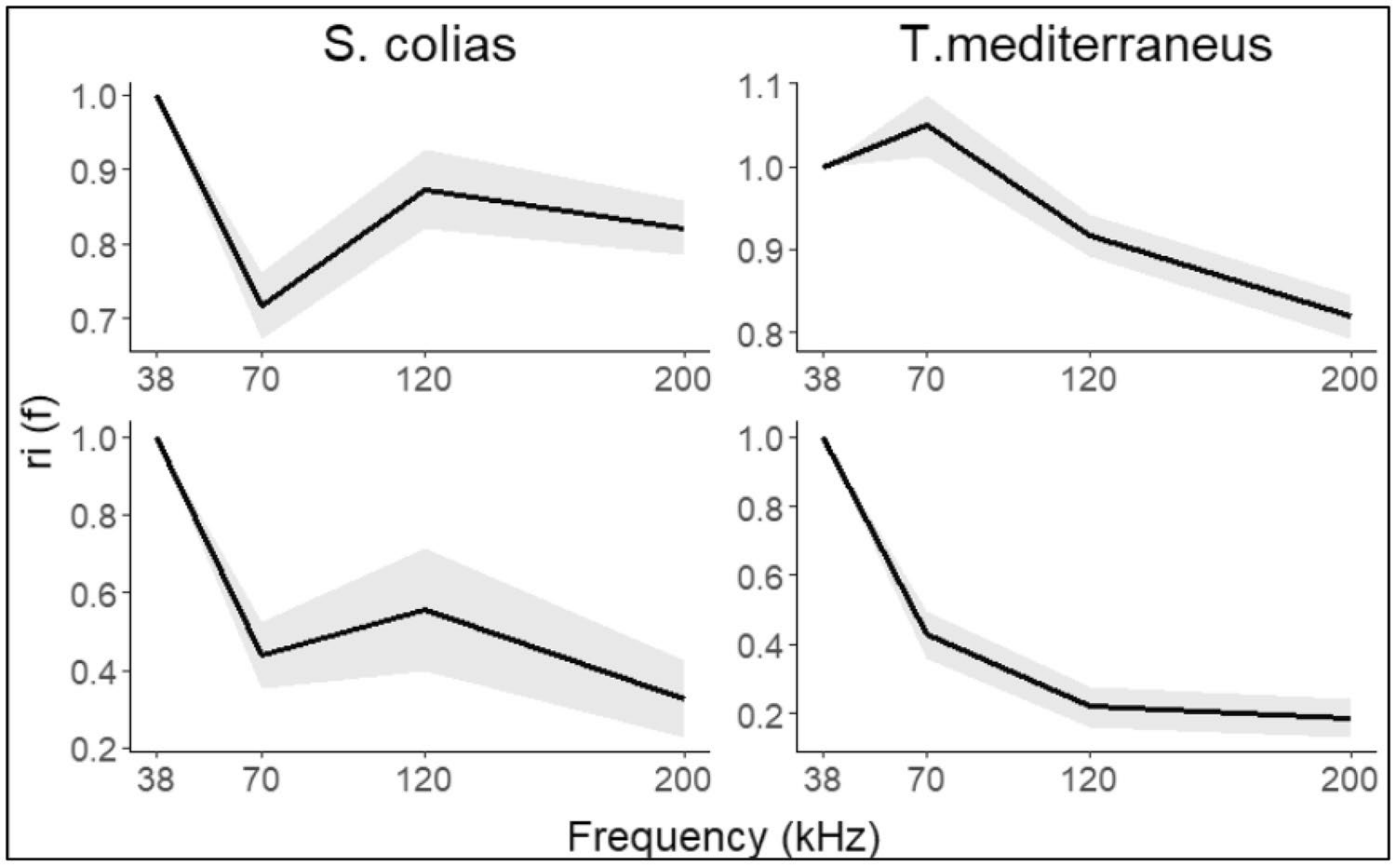
Mean relative frequencies responses (continuous lines) and confidence intervals (dashed areas) of *T. mediterraneus* and *S. colias* considering a mean tilt angle of 88°, standard deviation of 13° (upper panel) and broadside angle (lower panel).

Conversely, the coefficient of determination *r*^2^ shows a good fit in the regression models with no differences between the slope fitted to the data and a fixed slope of 20. Figure 7 shows the relationship between TS and TL considering a tilt angle of 88° ± 13°. The higher standard error found for *Scomber colias* is mainly due to the lower number of samples. Thereafter, the overall mean ${\sigma }_{bs}$ obtained at discrete frequencies 70, 120 and 200 kHz were divided by the values resulting from 38 kHz getting the patterns shown in Fig. 8. At a tilt angle of 88° ± 13°, the ${r}_{i} (f)$ at 70 and 200 kHz settles at a ratio of ~ 0.35 and ~ 0.20 respectively for both species, whereas the main contrast concerns the results at 120 kHz, characterized by a difference of 0.30.

At a broadside angle, as expected, the mean backscattering cross-section values at the other frequencies rose, leading to all ${r}_{i} (f)$ values being over 0.7. In this case, the diagnostic frequency is 70 kHz, which is characterized by a ratio even higher than 1, rather than 120 kHz that yielded to similar ratios of ~ 0.90 for chub mackerel as well as for the Mediterranean horse mackerel. However, the trend between these frequencies showed a clear downward pattern for *T. mediterraneus* and an upward pattern for *S. colias* at the tilt angles taken into account.

## Discussion

The high amount of samples with intact swimbladder (77) achieved after the X-ray scans prove the validity of the experimental plan both on *T. mediterraneus* and *S. colias,* which was based on the best literature on this topic^24,43,46,48,49^. The X-ray scan revealed wide differences in the swimbladder morphologies and a slight variation in the orientation within the fish body, which was partially reflected in the backscattering variation between the two species. Nevertheless, the dorsal aspect is known to be the most important diagnostic character in TS studies^50^, and in our case, the dimensionless results showed similar mean-cross-sectional area values, despite the dorsal area of *T. mediterraneus* being slightly, but significantly, smaller. This can explain the closeness between *b*_20_ values which are within 2.5 dB of variation at any tilt angle cases (see Table 3). The cross-sectional area is also a fundamental parameter for the decision of the most correct TS-TL function, especially in our case where the other dimensionless swimbladder measurements were highly divergent (see Fig. 4). As demonstrated by McClatchie et al.^51^, the choice of correct slope for the species-specific TS-TL function should not be done a-priori but should be based on morphological analysis, choosing the most appropriate value^52^. For both the Mediterranean horse mackerel and the chub mackerel the mean cross-sectional area grows proportionally to the total length as shown in Fig. 2. Notably, the growth is allometric, since it significantly differs from the isometric regression (slope = 2.69, p < 0.001 for *T. mediterraneus*; slope = 2.6, p < 0.001 for, *S. colias*) see Figure S5.Therefore, assuming a slope value close to 20 cannot be considered a good approximation for the species considered in the present work. Nevertheless, in the following discussion, we will focus on the *b*_20_ results rather than on *b* for comparison purposes with other studies.

This is the second study concerning the use of backscattering models on fish species in the Mediterranean Sea; the first one described a model-based TS-TL relationship on *T. mediterraneus* and *S. colias*^39^. An advantage in modelling compared to empirical experiments is the possibility of increasing the amount of data when a limited number of samples are available, gaining species-specific TS variability and backscattering patterns^53,54^. Approximate analytical models such as KRM are a good trade-off between model complexity and computational demand, which limits the application of numerical models such as the FEM model to relatively smaller-filled objects^55^, even though using an approximation model involves a decrease in accuracy especially at steeper angles and low aspect ratio^45^. Therefore, an accuracy test could be useful to determine the suitability of the model based on species and swimbladder morphologies. Nevertheless, the KRM model applied herein sums coherently the backscatter from fish body and swimbladder which is not always the case in nature. The inhomogeneous structure of fish and their variable orientation could yield different echo phases. Consequently, the scatter distribution can change rapidly, leading to more complicated variations in the echo amplitude during acoustic data collection in the field. In the present work, a FEM model was selected as a benchmark since using the formal exact theoretical solution to simulate fish scattering from a Prolate Spheroid Model (PSM) presents certain computational difficulties when calculating the infinite series, particularly when dealing with large aspect ratio values. In contrast, employing a Finite Element Method (FEM) model makes this process simpler. Moreover, FEM backscatter has been demonstrated to have negligible differences with analytical Prolate Spheroid model (PSMS) at all angles and aspect ratios^45^. To apply the KRM model, Jech et al.^20^ suggested dividing the fish body and the swimbladder into finite cylinders 1 mm thick for operational purposes, while Macaulay et al.^45^ used very thin cylinders up to 0.05 mm. However, slicing real shapes under ʎ/10 accuracy is in many instances time-consuming, hence we tried to test the accuracy of the model by considering coarser measurements. In the case of fish body, it did not affect the TS results likely due to the fairly small variation of the object along the x axes. Conversely, the slight differences detected for the swimbladder could affect the backscatter. Nevertheless, considering the whole fish and the results obtained on a theoretical sphere, a slice thickness of 2 mm appears not suitable, while 1 mm size can be considered highly accurate and any further effort in obtaining tiny cylinder unnecessary. We, accordingly, suggest collecting a measure1 mm each both for swimbladder and for fish body for use of the KRM model. Moreover, the comparison between FEM and the approximate analytical model proved the suitability of KRM for our swimbladder shapes. Indeed, the maximum difference of 1 dB detected between them on an elongated swimbladder characterized by a semi-minor axes less than half of the length of the semi-major axes can be considered a good approximation^45^.

New TS-TL relationships derived from models are seldom employed for acoustic abundance estimates^53^. Most frequently, models are exploited for comparison purposes with ex-situ or in-situ experiments^32,33,56,57^. The relationship found in this work can be compared with the study carried out on these species by Palermino et al.^58^. During these ex-situ experiments, the tilt angles of the specimens were not detected, the choice of a wide range of tilt angles for model development, therefore, can help the interpretation of both model and experimental data.

In the previous study, Palermino et al.^58^ found *b* and *b*_20_ values for the two species very close to each other as reported in Table 4. Our findings at an assumed normal swimming behavior of 88° ± 13° were ~ 5 dB higher for both species and along the tilt angle intervals, the difference is almost constant except for orientations of 90° ± 20° and 101° ± 12°, where the results are lower and closer to the *b*_20_ = − 71.4 dB for *T. mediterraneus* and *b*_20_ = − 71.6 dB for *S. colias* obtained in Palermino et al.^58^ (Table 4). Fish orientation is one of the parameters that influence the TS the most, especially when it is measured in unnatural and natural conditions^13,15,46,59^. It has been demonstrated that during ex-situ experiments fishes display a steeper angle than in their natural state which in turn can affect TS measurements^60^. Despite the efforts and the novelty of the use of a piece of rope instead of a hook performed during the single ex-situ experiments conducted in 2013 and 2014^58^, the Mediterranean horse mackerel and chub mackerel specimens were likely constrained to a fairly abnormal swimming behavior. Consequently, at 38 kHz the *b*_20_ value of − 70.4 dB for *T. mediterraneus* and − 69.43 dB for *S. colias* found through the KRM model considering an abnormal tilt angle displacement in this study could be considered almost in agreement with empirical experiments within a 2 dB interval. We can assume these results as being the correct ones also for the other tilt angle intervals with an approximation of 2 dB. Moreover, the fish backbone backscatter could have caused shadowing effects on the swimbladder, due to the dorsal configuration, leading to differences in TS values measured from the ex situ experiment compared to ones computed through the KRM model in which the fish backbones were not modelled^61^. Nevertheless, in swimbladder fish, this effect could be more relevant for higher frequencies than for lower and we assume no significant differences at 38 kHz^61,62^.

**Table 4:** Comparison between conversion parameters b and b_20_ found in this work and the results obtained in Palermino et al.^58^ at 38 kHz.

Tilt angle (°)	*T. mediterraneus*				*S. colias*			
	Present work		Ex-situ experiment		Present work		Ex-situ experiment	
	*b*	*b*_20_	*b*	*b*_20_	*b*	*b*_20_	*b*	*b*_20_
90 (s.d = 5)	− 65.88	− 64.27	− 64.9	− 71.4	− 71.25	− 66.00	− 63.8	− 71.6
90 (s.d = 10)	− 70.12	− 64.51			− 73.2	− 66.40		
90 (s.d = 20)	− 71.58	− 65.48			− 74.06	− 67.58		
101 (s.d = 12)	− 56.52	− 70.40			− 62.54	− 69.13		
88 (s.d = 13)	− 71.65	− 64.40			− 74.48	− 66.65		

The reported values are in dB re 1 m^2^. Note the shortfalls that underline the b_20_ values usually employed for the conversion of backscattering volume in biomass.

Theoretical and empirical experiments proved that the peak of backscatter in fish with swimbladder is achieved at a negative tilt angle of ~ 10°, followed by a drop at steeper angles characterized by individual-fish pattern^35,53,56^. *S. colias* displays a TS pattern against tilt angle characterized by modest variation along the tilt angles reaching a peak at around − 4° at 38 kHz. Conversely, the TS peak of *T. mediterraneus* was recorded close to − 10° at the same frequency^39^. Some authors provided evidence of a quite steeper positive or negative tilt angle kept by fish of the *Scomber* and *Trachurus* genus in normal swimming behaviour^63,64^. It should be highlighted that when the vessel approach fish schools or shoals during daytime acoustic surveys activity they are inclined to display an avoidance behaviour, especially in shallow waters causing an abnormal swimming behaviour leading in turn to a drop in the TS^65,66^. However, fish orientation depends on an ensemble of natural factors such as light intensity and feeding migrations^63,64,67^, and therefore the most suitable *b*_20_ value could vary between shifting survey conditions. The *b*_20_ values presented in Tables 3 and 4 at normal swimming behaviour (88° ± 13°) are closer to that of − 68.7 dB now in use in the Mediterranean Sea^68,69^. They are also close to the values detected by other studies conducted in the Atlantic and the Pacific Oceans on related species: *Trachurus capensis*, *Trachurus symmetricus mutphy* and *Scomber japonicus*^65,70–72^ Conversely, they diverge from other studies carried out on these species^35,70,73,74^. Notably, the conversion parameter values obtained with a tilt angle of 101° ± 12° for *T. mediterraneus* here are closer to the results published by Peña and Foote^35^ that applied a Kirchhoff approximation on specimens of *Trachurus symmetricus murphy* subjected to MRI scanner.

The small differences could be mainly linked to the species but also to the methodology and the size of the fish. The multi-frequency approach for the identification of fish species has been applied since the early 2000s^75–77^. It is now commonly used in post-processing acoustic data analysis worldwide^78^. However, the technique is still focused on a few target species due to the lack of data on other pelagic fish species especially in the Mediterranean Sea^79^.

The results obtained in this study applying the relative frequency response formula pointed out a clear opposite pattern between 70 and 120 kHz, which is not affected by the tilt angle: *S. colias* showed a rising curve while *T. mediterraneus* displayed a decreasing curve. These are the first multi-frequency backscatter evidence on both species worldwide, although the relative frequency response of the congeneric species *Trachurus trachurus* has already been studied in 2005^80^. Our results differ from the latter, giving the possibility to distinguish between the three co-occurrence species in the Adriatic Sea via an acoustic tool. Again, the typical swimbladder morphologies could be responsible for these acoustic fingerprints. The differences in swimbladder physiology and morphological structures could justify the change in RFI between species belonging to different genera. Conversely, the variability in condition factors and habitat preferences could justify differences in RFI between congeneric species^81^. Moreover, the technological improvements undertaken by acoustic equipment during the last two decades should be underlined, as they might have determined slight differences in TS values compared to past measurements. The current use of split-beam transducer enhances the detection of the precise position of fish in the three-dimensional space being particularly suitable for this kind of study^40^. In a broadband view, small variations in swimbladder shape translate into changes in the TS curve. This grant the opportunity to distinguish between species overcoming the influence of size^30,82,83^. The frequency-dependent backscatter depicted in Fig. 8 for a broadside angle reflects the broadband trend reported by Palermino et al.^39^, lending robustness to our analysis.

## Conclusions

Backscatter models are not intended to replace empirical measurements, but they are useful to corroborate target strength experiments and to fill the gap in the knowledge of species that hardly fulfil the requirements of monospecific shoals of spread fish for the application of the in-situ method. This is the case of *T. mediterraneus* and *S. colias* in the Mediterranean Sea. The implementation of a backscattering model added essential information to the knowledge of the acoustic reflectivity of *T. mediterraneus* and *S. colias* in the Mediterranean Sea. Through swimbladder measurements, we proved the allometric growth of the mean cross-section area for both species which supports the use of best fit *b* instead of *b*_20_ during the conversion of acoustic backscatter volume in abundance for the species this study deals with. The results provided underline the importance of the use of multiple frequencies, confirming that the higher the number of frequencies in use, the higher the species discrimination power gained. The results are in agreement with empirical measurements obtained during some ex-situ experiments carried out in the Adriatic Sea. Nevertheless, further effort should be made to get in-situ measures of TS of ancillary species, since during ex-situ experiments the swimming behaviour may not be representative of the natural state of fish.

### Supplementary Information

Supplementary Information.

## Data Availability

Data available on request. The data underlying this article will be shared on reasonable request to the corresponding author.
