# Supplementary material for: Application of an analytical approach to characterize the target strength of ancillary pelagic fish species
Source: Sci Rep. 2023 Sep 13;13:15182. doi: 10.1038/s41598-023-42326-4 (PMC10499918; doi:10.1038/s41598-023-42326-4)
Supplement: Supplementary file 1 — Supplementary Information. [file 41598_2023_42326_MOESM1_ESM.pdf]

Application of an analytical approach to characterize the target strength of ancillary pelagic fish species

Antonio Palermino, Andrea De Felice, Giovanni Canduci, Ilaria Biagiotti, Ilaria Costantini, Michele Centurelli, Iole Leonori

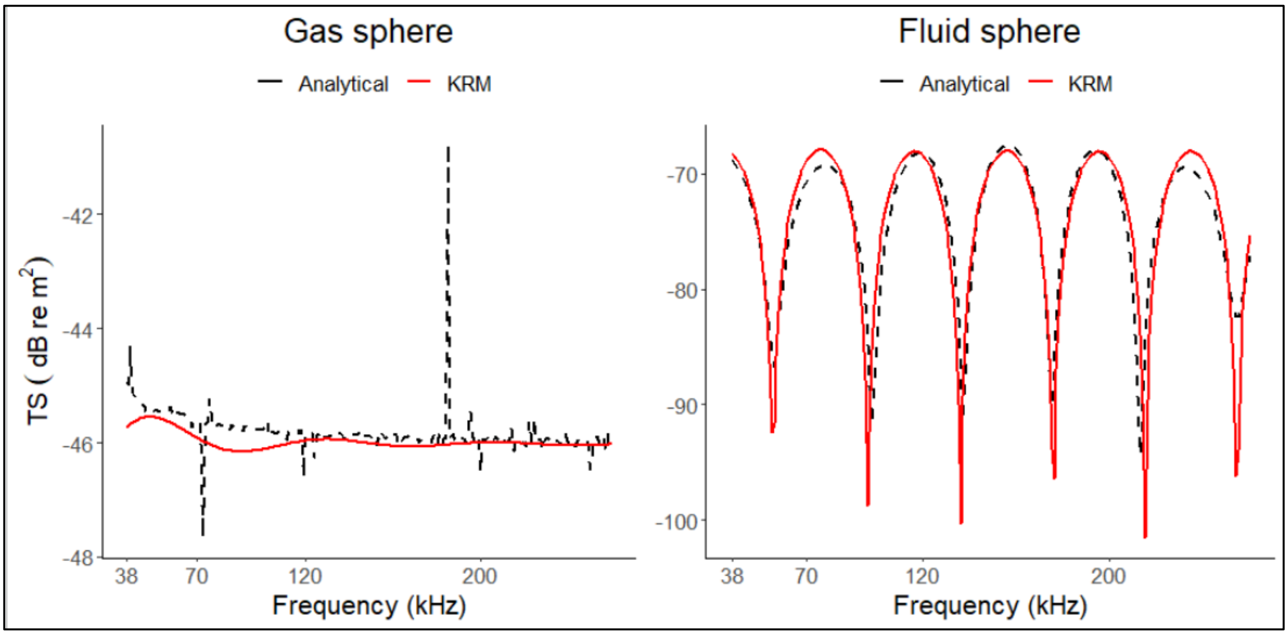

Figure S1. Comparison between analytical model (black) and KRM model (red) on a sphere of 10 mm radius in broadband spectra

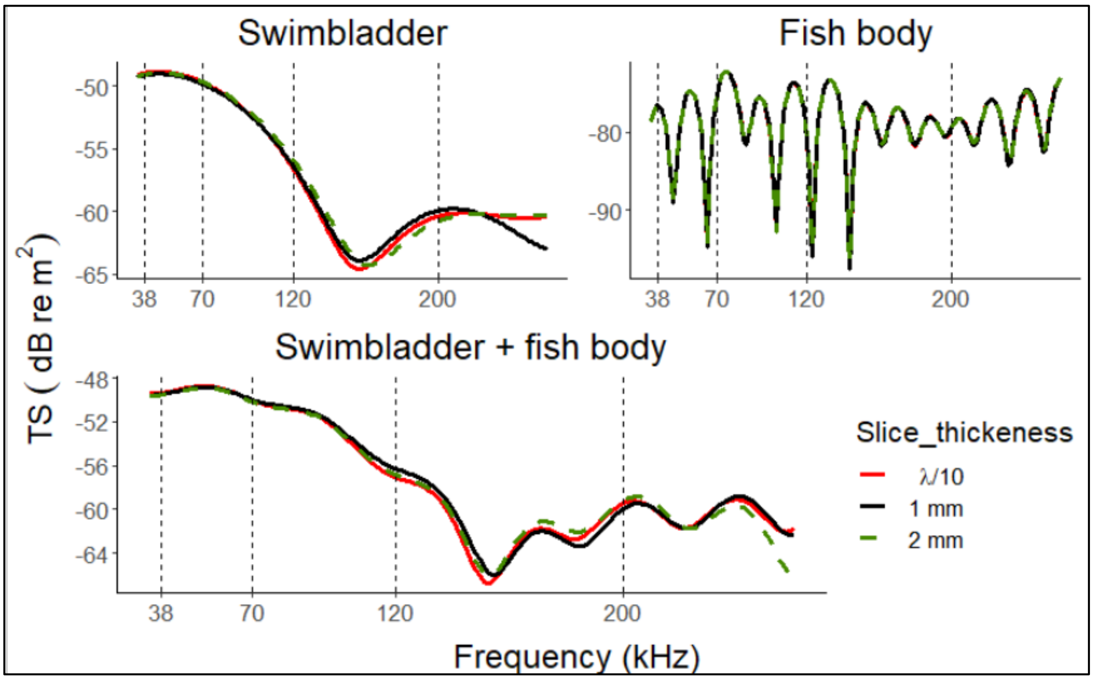

Figure S2. Broadband backscatter comparison between coarse and refine slice thickness on a *S. colias* specimen of 11.5 cm total length considering a tilt angle of + 25°

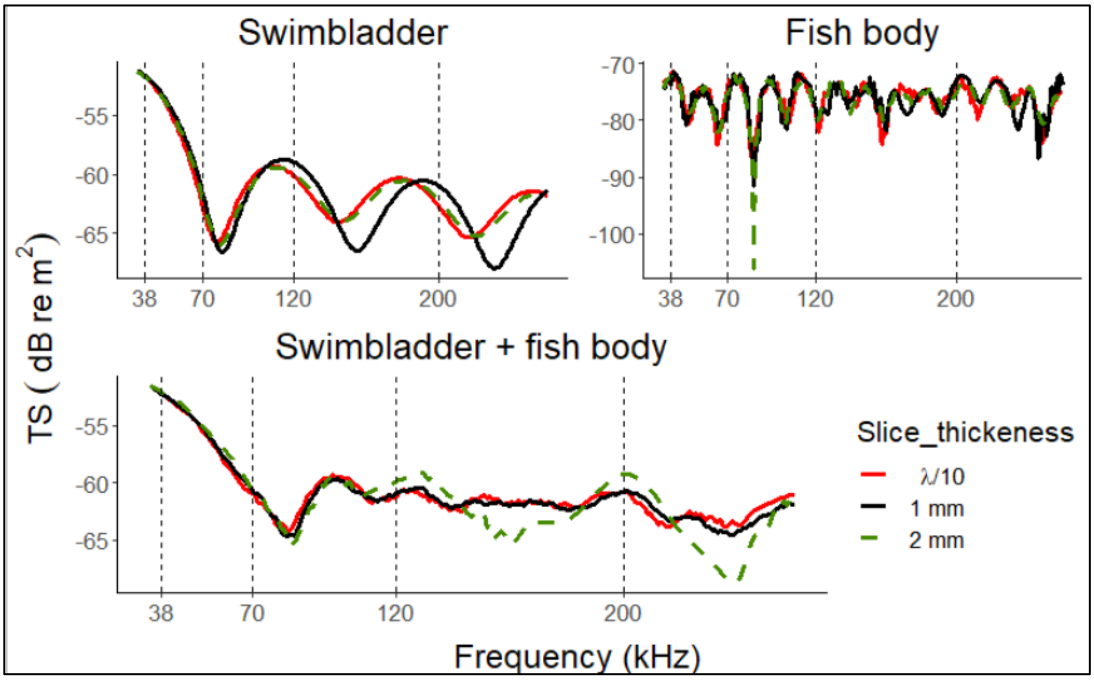

Figure S3. Broadband backscatter comparison between coarse and refine slice thickness on a *S. colias* specimen of 11.5 cm total length considering a tilt angle of - 25°

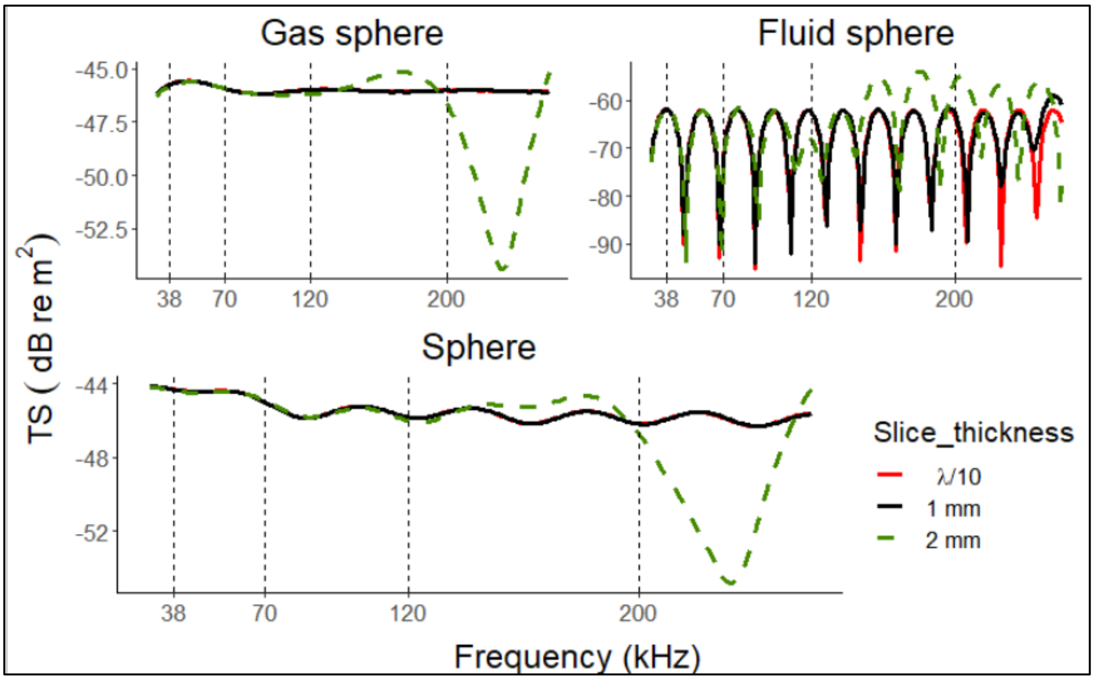

Figure S4. Broadband backscatter comparison between coarse and refine slice thickness on a theoretical gas filled sphere of 10 mm radius (top left), fluid filled sphere of 20 mm radius (top right) and the summation of both (below).

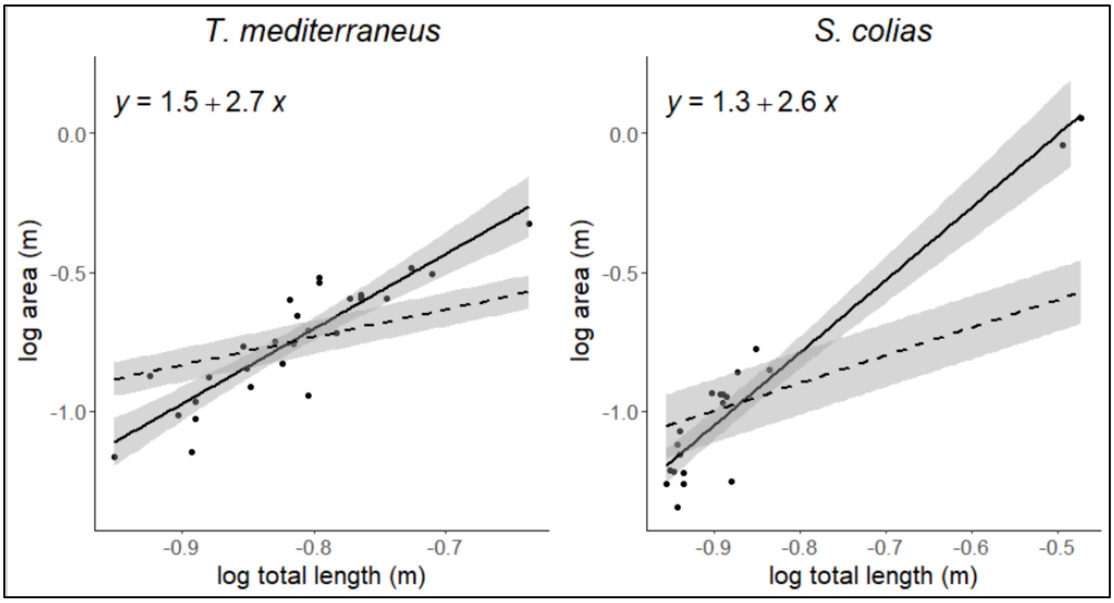

Figure S5. In continuous lines the best fit allometric relationship between the log of mean cross-sectional area of the swimbladder (y axis) and log of fish length (x axis). The dashed lines show the theoretical isometric relationship (with slope = 1). The allometric regression model equations are reported. The shadow areas express the 95% confidence intervals.

FEM model brief description and formula:

The FEM model adapted from acoustic scattering off an ellipsoid, COMSOL MULTIPHYSICS 5.6, Acoustics Module model Library) was implemented using COMSOL Multiphysics (v.6) (COMSOL, 2021), where The Kirchhoff - Helmholtz integral equation was solved in the frequency domain for each small element computing the far field backscattered pressure, as (Ivansson, 2017):

$$P_{ext}(R) = -\frac{1}{4\pi}\int_{S(r)} e^{\frac{ik(r\,R)}{|R|}}\left(\nabla p(r) - ikp(r)\frac{R}{|R|}\right)(-n)\,dS$$

where the incident pressure field was set at 1 Pa. The exterior field computation was set at 1 m.The mesh resolution was set to at least 10 meshes per wavelength at the highest considered frequency (200 kHz). The same acoustic parameters presented in Table 1 were applied for the computations.

Successively TS was computed using the following formula:

$TS = 20log10(\sigma_{bs}(P_{ext}(R)(P_{ext}(R)z\ axis\ , P_{ext}(R)y\ axis, P_{ext}(R)x\ axis)))$ [dB re 1 m^2]

where (σ_{bs}) [m^2] is the backscattering cross-section of a single target, x axis directivity = Cos(θ), y axis directivity = Sin(θ)*cos(Φ), z axis directivity = Sin(θ)*sin(Φ) where θ=90° and Φ=90°. The same KRM acoustic parameters were set.
